# No Effect of Folic Acid Supplementation on Global DNA Methylation in Men and Women with Moderately Elevated Homocysteine

**DOI:** 10.1371/journal.pone.0024976

**Published:** 2011-09-23

**Authors:** Audrey Y. Jung, Yvo Smulders, Petra Verhoef, Frans J. Kok, Henk Blom, Robert M. Kok, Ellen Kampman, Jane Durga

**Affiliations:** 1 Department of Epidemiology, Biostatistics, and HTA, Radboud University Nijmegen Medical Center, Nijmegen, The Netherlands; 2 Department of Internal Medicine and Metabolic Unit, VU University Medical Center, Amsterdam, The Netherlands; 3 Institute for Cardiovascular Research IcaR, VU University Medical Center, Amsterdam, The Netherlands; 4 Division of Human Nutrition, Wageningen University, Wageningen, The Netherlands; 5 Unilever Research and Development, Vlaardingen, The Netherlands; 6 Top Institute Food and Nutrition, Wageningen, The Netherlands; 7 Cognitive Sciences Group, Nutrition & Health Department, Nestlé Research Center, Lausanne, Switzerland; Brigham & Women's Hospital, and Harvard Medical School, United States of America

## Abstract

A global loss of cytosine methylation in DNA has been implicated in a wide range of diseases. There is growing evidence that modifications in DNA methylation can be brought about by altering the intake of methyl donors such as folate. We examined whether long-term daily supplementation with 0.8 mg of folic acid would increase global DNA methylation compared with placebo in individuals with elevated plasma homocysteine. We also investigated if these effects were modified by MTHFR C677T genotype. Two hundred sixteen participants out of 818 subjects who had participated in a randomized double-blind placebo-controlled trial were selected, pre-stratified on MTHFR C677T genotype and matched on age and smoking status. They were allocated to receive either folic acid (0.8 mg/d; n = 105) or placebo treatment (n = 111) for three years. Peripheral blood leukocyte DNA methylation and serum and erythrocyte folate were assessed. Global DNA methylation was measured using liquid chromatography-tandem mass spectrometry and expressed as a percentage of 5-methylcytosines versus the total number of cytosine. There was no difference in global DNA methylation between those randomized to folic acid and those in the placebo group (difference = 0.008, 95%CI = −0.05,0.07, P = 0.79). There was also no difference between treatment groups when we stratified for MTHFR C677T genotype (CC, n = 76; CT, n = 70; TT, n = 70), baseline erythrocyte folate status or baseline DNA methylation levels. In moderately hyperhomocysteinemic men and women, long-term folic acid supplementation does not increase global DNA methylation in peripheral blood leukocytes.

ClinicalTrials.gov NCT00110604

## Introduction

Disturbances in DNA methylation, one of several epigenetic mechanisms, have been implicated in many diseases ranging from cancers to cardiovascular disease. Two main types of changes in DNA methylation patterns have been observed – global hypomethylation [1], and regional hypermethylation, particularly in the promoter regions of certain genes such as tumor suppressor genes or imprinted genes [2], [3], [4]. Global DNA methylation provides genomic stability and structure [5] while promoter hypermethylation inhibits the transcription of associated genes, resulting in gene silencing [6].

Folate-mediated one-carbon metabolism is key for DNA methylation as well as for nucleotide synthesis, and DNA repair and stability. Folate, a water soluble B-vitamin, functions as a donor and acceptor of one-carbon units in its various forms in cellular metabolism. As such, manipulation of intake of folate or other factors involved in one-carbon metabolism can largely influence DNA methylation, and methyl-deficient and repletion diets have been shown to alter global DNA methylation patterns in animal studies [7] and also in human studies [8], [9] although the evidence is not unequivocal [10], [11].

Many factors affect the availability of methyl groups in folate-mediated one-carbon metabolism. One of the most extensively studied genetic polymorphisms which can modify folate metabolism is the 677C→T polymorphism of the gene that codes for the enzyme methylenetetrahydrofolate reductase (MTHFR). Methylenetetrahydrofolate reductase converts 5,10-methylenetetrahydrofolate to 5-methyltetrahydrofolate, which is required for the conversion of homocysteine to methionine. Rates of folate metabolism have been shown to differ between those who have the TT genotype compared to those with the CC genotype; production of methyl group donors is higher in those with the CC genotype compared to those with the TT genotype [12].

Indeed, on a functional level, since homocysteine has to be remethylated to form methionine, individuals with the MTHFR 677TT polymorphism have higher concentrations of plasma homocysteine when folate status is low compared to those with the other two genotypes [13], [14]. MTHFR 677TT individuals were also found to have lowered DNA methylation in their leukocytes when folate status is low compared to those who have the MTHFR 677CC genotype [15].

Given the importance of DNA methylation in epigenetic regulation, and the documented effects of experimental folate deprivation and repletion, it is relevant to know the ways in which folic acid supplementation impacts DNA methylation. We investigated whether daily supplementation of 800 µg folic acid for 3 years would increase global DNA methylation in peripheral blood leukocytes compared with placebo in individuals with elevated homocysteine concentrations. In addition, we addressed effects separately in strata of the MTHFR C677T genotypes.

## Materials and Methods

### Ethics Statement

The study protocol was approved by the Medical Ethics Committee of Wageningen University. All participants gave written informed consent.

### Study Participants

Participants were a subset of Dutch men and post-menopausal women aged 50–70 years from a central-eastern region of the Netherlands and who had participated in the Folic Acid and Carotid Intima-media Thickness (FACIT) trial [16]. The FACIT trial was designed to investigate the effect of folic acid supplementation on atherosclerotic progression. Additional outcomes of the trial were age-related decline in cognitive function and hearing [17], [18], [19]. Here we present data for the effect of folic acid on global DNA methylation for a subset of participants.

Detailed participant recruitment for this randomized double blind placebo-controlled trial has been described previously [17]. Briefly, participants were recruited using municipal and blood-bank registries and randomized between November 1999 and April 2001. Inclusion criteria were plasma total homocysteine concentrations between 13 µmol/L and 26 µmol/L, vitamin B12 less than 200 pmol/L, no intestinal disease, and no current use of B-vitamin supplements or other medications that could influence folate metabolism or atherosclerotic progression. Finally, more than 80% self-reported compliance during a 6-week placebo run-in period was required.

After the initial measurement sessions, participants were randomly allocated to treatment of 800 µg folic acid per day or placebo with permuted blocks of sizes four and six, which varied randomly. Specialized staff who were not involved in the study allocated and labeled the capsule boxes with participants' unique sequence number. Those participants in the same household received the same treatment. The folic acid and placebo capsules were produced by Swiss-Caps Benelux (Heerhugowaard, Netherlands) and were identical in appearance. Capsules were individually packaged in foil strips containing 28 capsules per strip with the days of the week printed on the back. Every year, participants received a 13-month supply of capsules. Compliance was determined by capsule-return counts and a diary that registered missed capsules. Diaries and capsules were returned by participants every 12 weeks.

For the present study, changes in global DNA methylation were assessed from stored frozen aliquots from a sub-sample of the FACIT trial participants. Using only the data from the participants who had completed the study (n = 818), participants in the folic acid arm of the trial were pre-stratified by MTHFR C677T genotype and matched to participants in the placebo arm on age and current smoking status. Subjects were matched on these variables because they are thought to influence global DNA methylation [20], [21], [22], [23], [24], [25]. Additionally, sampling was done such that each group of MTHFR C677T genotype was of the same size. This was done to increase our power of hypotheses testing related to the genotypes and their effects on global DNA methylation. Initially, 120 folic acid participants were matched to 120 in the placebo group, but 24 samples were not measured due to human error in sample retrieval.

### Laboratory Measurements

Fasting venous blood was processed and samples were stored at −80°C. We measured serum folate, erythrocyte folate, serum vitamin B12, plasma total homocysteine, plasma vitamin B6 (pyridoxal 5′-phosphate (PLP)), in addition to serum creatinine and lipids as described elsewhere [16]. In brief, serum and erythrocyte folate and serum vitamin B12 were measured using a chemiluminescent immunoassay (Immulite® 2000, diagnostic Products Corporation). Erythrocyte folate measurements were done in duplicate. Plasma homocysteine was determined with high performance liquid chromatography (HPLC) and fluorimetric detection [26]. Vitamin B6 was measured by HPLC. DNA was extracted from blood leukocytes and the C677T polymorphism in the methylenetetrahydrofolate reductase (MTHFR) gene was determined by PCR and restriction digest with *Hin*F1 and attained at the beginning of the study. Intra- and inter-assay variation coefficients for laboratory analyses were <15%. A validated food frequency questionnaire was used at baseline and after the intervention to estimate dietary folate intake during the past 3 months [27].

Quantification of global DNA methylation was measured by liquid chromatography-tandem mass spectrometry [28]. Global DNA methylation is expressed as a percentage of 5-methylcytosines versus the total number of cytosines present in the genome.

### Statistical Methods

Non-parametric tests were used to compare differences within groups and between groups before and after the intervention. Analysis of covariance (ANCOVA) was used to estimate differences between groups at baseline and after the intervention and also to compare the effect of supplementation on global DNA methylation adjusted for baseline DNA methylation and covariates. Medians, inter-quartile ranges, and 95% confidence intervals are presented. The variables on which subjects were matched (i.e. age and current smoking status) were used as covariates in our model. We performed subgroup analyses for those with deficient to low-normal erythrocyte folate status at baseline and those with low and high baseline DNA methylation. We also tested if the effect of folic acid on global DNA methylation was modified by MTHFR C677T genotype, age, current smoking status, alcohol intake, body mass index (BMI), and sex. Previous literature suggests that BMI may be associated with global DNA methylation [29], [30] and sex has been shown to influence DNA methylation [31], [32], [33]. There was no missing data on any of our outcome variables. Differences were considered significant at P<0.05. All analyses were computed using SAS version 9.2 (SAS Institute Inc., Cary, NC).

## Results

Two hundred sixteen participants (folic acid n = 105 and placebo n = 111) were included in our analyses. Table 1 depicts the baseline characteristics of the population. There were slightly more men in the placebo group than in the folic acid group (66.7% vs. 62.9%), and more current smokers in the folic acid group (16.2% vs. 13.5%). Concordant with the stratification procedure, there were roughly equal numbers of people in each MTHFR C677T genotype for both treatment groups. Folate intake was similar between the treatment groups, but alcohol consumption was higher in the folic acid group. Overall, participants had a low median folate intake (188 µg/day), but there were only 20 serum folate deficient (≤7 nmol/L) individuals and five individuals, who were deficient based on erythrocyte folate concentrations (≤305 nmol/L).

**Table 1:** Baseline characteristics of the population.

	Folic acid (n = 105)	Placebo (n = 111)
**Demographics**		
Age (years), mean (standard deviation)	60.9 (5.4)	60.9 (5.5)
Sex, male (%)	66 (62.9%)	74 (66.7%)
Education (high, middle, low), n (%)	High = 32 (30.5%), middle = 41 (39.1%), low = 32 (30.5%)	High = 25 (22.5%), middle = 43 (38.7%), low = 43 (38.7%)
**Dietary factors**		
Alcohol intake, g/day, median (inter-quartile range)	14.5 (5.5–24.4)	10.3 (3.5–22.0)
Folate intake, µg/day, median ( inter-quartile range )	185.8 (159.7–220.7)	194.3 (156.0–236.4)
Serum vitamin B12, pmol/L, median ( inter-quartile range )	295 (259–359)	283 (247–374)
Serum vitamin B6, nmol/L, median ( inter-quartile range )	31.8 (25.5–41.4)	31.4 (23.7–44.5)
**Non-dietary factors**		
BMI, mean (standard deviation)	26.9 (3.7)	26.4 (4.2)
Current smokers, n (%)	17 (16.2%)	15 (13.5%)
MTHFR C677T genotype, n (%)	CC: 36 (34.3%), CT: 36 (34.3%), TT: 33 (31.4%)	CC: 40 (36.0), CT: 34 (30.6%), TT: 37 (33.3%)

Table 2 shows the effects of folic acid supplementation on several metabolites throughout the study. As expected, there were large increases in serum folate (535% increase), and erythrocyte folate (207% increase), and a fall in plasma total homocysteine (21.6% decrease) after the intervention in the folic acid group compared to placebo (*P*<0.0001 for all). There were no changes in dietary folate intake during the course of the intervention.

**Table 2:** Biochemical measurements throughout the study.

Metabolite	Folic acid (n = 105)	Placebo (n = 111)	p-value
**Serum folate (nmol/L)**			
Baseline	12.0 (9.9–14.0)^1^	10.8 (8.5–13.9)	0.01
Year 3	76.3 (53.4–106.0)	12.5 (10.3–16.2)	<0.0001
**Erythrocyte folate (nmol/L)**			
Baseline	682.7 (528.8–895.8)	677.5 (548.2–807.6)	0.37
Year 3	2100.7 (1767.2–2611.2)	679.0 (564.6–864.3)	<0.0001
**Dietary folate intake (µg/day)**			
Baseline	185.8 (159.7–220.7)	194.3 (156.0–236.4)	0.48
Year 3	173.0 (152.0–197.0)	173.0 (146.0–209.0)	0.48
**Plasma total homocysteine (µmol/L)**			
Baseline	12.5 (11.3–14.5)	13.0 (11.4–15.5)	0.24
Year 3	9.8 (8.8–11.0)	13.4 (11.6–15.1)	<0.0001

1 values given are median (inter-quartile range).

Median global DNA methylation levels throughout the study can be found in table 3. There was no significant change in DNA methylation within treatment groups after three years (folic acid difference = 0.02, 95%CI = −0.05,0.02, *P* = 0.39; placebo difference = 0.02, 95%CI = −0.06,0.01, *P* = 0.62). There was also no difference in the effect of supplementation after three years between the two groups (difference = 0.008, 95%CI = −0.05,0.07, *P* = 0.79). There was no significant treatment effect of folic acid on global DNA methylation for those with less than normal baseline red blood cell (RBC) folate (≤500 nmol/L) (Table 3) when compared with the placebo group. Additionally, there was no trend towards higher methylation following supplementation in those with below median DNA methylation at baseline (median difference = 0.003, 95%CI = −0.06,0.07, *P* = 0.92) compared with placebo. When we stratified according to MTHFRC677T genotype, there was no effect of folic acid on global DNA methylation in any of the genotype subgroups (from Table 3, *P* = 0.65 for CC, *P* = 0.32 for CT, *P* = 0.45 for TT), nor did we observe an effect when we stratified according to alcohol intake (data not shown). Furthermore, for those who were both MTHFR677TT and having less than normal baseline RBC folate status, there was also no significant effect of folic acid on global DNA methylation compared with the placebo group (data not shown). In other subgroup analyses, there was no difference overall in global DNA methylation between males and females. Finally, no differences in global DNA methylation due to folic acid supplementation were observed when we stratified by age, current smoking status, body mass index (BMI), or sex (data not shown).

**Table 3:** Folic acid supplementation on global DNA methylation for the whole study population, for those with less than normal RBC folate status at baseline, low and high baseline methylation, and stratified by MTHFR C677T genotype.

Whole study population				
	Folic acid (n = 105)	Placebo (n = 111)	Difference (95%CI)	p-value
Baseline	4.63 (4.53,4.74)^1^^,^ 2	4.60 (4.51,4.72)	−0.01 (−0.07,0.05)	0.71
Year 3	4.62 (4.49,4.73)	4.56 (4.56,4.63)	0.004 (−0.06,0.07)	0.90
Difference (95%CI)	−0.02 (−0.05,0.02)	−0.02 (−0.06,0.01)	0.008 (−0.05,0.07)	0.79
p-value	0.39	0.62		

Deficient to low-normal RBC folate status (≤500 nmol/L)				
	Folic acid (n = 22)	Placebo (n = 22)	Difference (95%CI)	p-value
Baseline	4.62 (4.53,4.64)	4.59 (4.51,4.70)	0.039 (−0.13,0.21)	0.65
Year 3	4.59 (4.52,4.76)	4.69 (4.52,4.73)	−0.046 (−0.19,0.10)	0.53
Difference (95%CI)	0.01 (−0.09,0.10)	0.04 (−0.09,0.13)	−0.058 (−0.20,0.08)	0.41
p-value	0.81	0.80		

Low/high baseline methylation (median split)				
	Folic acid	Placebo	Difference (95%CI)	p-value
**Low <4.615**	n = 48	n = 60		
Baseline	4.51 (4.40,4.56)^1^^,^ 2	4.51 (4.42,4.57)	−0.002 (−0.05,0.04)	0.92
Year 3	4.53 (4.40,4.66)	4.54 (4.45,4.66)	0.003 (−0.06,0.07)	0.93
Difference (95%CI)	0.04 (−0.01,0.08)	0.02 (−0.02,0.08)	0.003 (−0.06,0.07)	0.92
p-value	0.045	0.045		
**High ≥4.615**	n = 57	n = 51		
Baseline	4.72 (4.66,4.82)	4.73 (4.66,4.89)	0.02 (−0.03,0.10)	0.29
Year 3	4.68 (4.59,4.78)	4.66 (4.54,4.78)	0.02 (−0.08,0.14)	0.63
Difference (95%CI)	−0.06 (−0.09,−0.03)	−0.08 (−0.14,−0.02)	0.008 (−0.10,0.11)	0.87
p-value	0.002	0.006		

MTHFR C677T genotype				
	Folic acid	Placebo	Difference (95%CI)	p-value
**CC**	n = 36	n = 40		
Baseline	4.63 (4.60,4.64)	4.60 (4.57,4.65)	−0.035 (−0.12,0.05)	0.41
Year 3	4.58 (4.49,4.69)	4.58 (4.51,4.63)	−0.035 (−0.12,0.05)	0.42
Difference (95%CI)	−0.07 (−0.13,−0.01)	−0.06 (−0.10,−0.01)	−0.017 (−0.09,0.06)	0.65
p-value	0.02	0.03		
**CT**	n = 36	n = 34		
Baseline	4.63 (4.56,4.69)	4.54 (4.47,4.64)	−0.06 (−0.16,0.04)	0.27
Year 3	4.63 (4.57,4.69)	4.66 (4.55,4.70)	0.06 (−0.07,0.19)	0.37
Difference (95%CI)	−0.01 (−0.06,0.04)	0.05 (0.00,0.13)	0.07 (−0.06,0.20)	0.32
p-value	0.78	0.10		
**TT**	n = 33	n = 37		
Baseline	4.64 (4.54,4.71)	4.63 (4.58,4.70)	0.06 (−0.05,0.18)	0.29
Year 3	4.62 (4.56,4.72)	4.59 (4.54,4.64)	−0.01 (−0.14,0.12)	0.86
Difference (95%CI)	0.02 (−0.03,0.10)	−0.04 (−0.09,0.04)	−0.04 (−0.16,0.07)	0.45
p-value	0.32	0.61		

1 values are given as median (inter-quartile range).

2 global DNA methylation is expressed as a percentage of 5-methylcytosines versus the total number of cytosines present in the genome.

## Discussion

In this double-blind randomized controlled trial of daily folic acid supplementation in a Dutch population with moderately elevated homocysteine, we did not find any evidence of changes in global DNA methylation after three years of supplementation. Those whose genotype has previously been associated with lower leukocyte DNA methylation, were no exception.

To our knowledge, seven randomized controlled trials have previously examined the effect of folic acid on global DNA methylation (Table 4). Two of these trials were also performed in cancer-free populations, but were short-term (12 weeks duration), used supraphysiological doses of folic acid (2 mg folic acid and 20 µg vitamin B12 and 1.2 mg folic acid, respectively), and used small sample sizes (included 63 and 61 volunteers, respectively [34], [35]. No effect of folic acid on global DNA methylation in leukocytes was observed in both these studies, which used [^3^H] methylation incorporation to assess DNA methylation. Additionally, five randomized controlled trials in cancer-related studies were performed, as a global loss of methylated cytosines is frequently observed in cancers [36] and continues to be an active area of research. Two of these studies reported null results [37], [38] and three studies, all of which were carried out in populations with colorectal carcinomas or adenomas, noted an increase in global DNA methylation following daily administration with 10 mg, 5 mg, and 400 µg folic acid, respectively [39], [40], [41]. In these studies, global DNA methylation was assessed only in colorectal tissues with the exception of one study that also measured global DNA methylation in leukocytes in addition to colonic mucosa [41]. In the latter study, there was a slight increase in global DNA methylation in peripheral blood leukocytes of individuals taking folic acid compared to those in the placebo group (P = 0.05), but not in colonic mucosa of colorectal adenoma patients following 400 µg daily folic acid supplementation for 10 weeks. Their study was conducted in colorectal adenoma patients, in whom folic acid metabolism may already be altered, subsequently affecting DNA methylation, which may explain for our differences in results. Our findings are aligned with those in other trials with cancer-free volunteers.

**Table 4:** Overview of all randomized controlled trials of folic acid with global DNA methylation as an endpoint.

CANCER-FREE POPULATION								
Study	Number of participants	Dose	Duration	Endpoint	DNA methylation assessment method	Baseline concentrations of folate^1^	Baseline levels of DNA methylation	Treatment effect/Outcome
Fenech *et al.* 1998 [34]	63 volunteers	2 mg folic acid and 20 µg vitamin B12	12 weeks	Global DNA methylation in leukocytes	[^3^H] methyl incorporation	RBC folate (nmol/L)Men 440.0 (20.4); women 363.8 (17.2)	Intervention 195200 dpm; placebo 169600 dpm	No effect
Basten *et al.* 2006 [35]	61 healthy volunteers	1.2 mg folic acid	12 weeks	Global DNA methylation in lymphocytes	[^3^H] methyl incorporation	RBC folate (nmol/L) intervention 552 (469–655), placebo 668 (508–796) [48]	Intervention 17508 dpm; placebo 16099 dpm	No effect
Present study	216 healthy volunteers (elevated Hcy)	800 µg folic acid	3 years	Global DNA methylation in leukocytes	Liquid chromatography-tandem mass spectrometry (LC-ES MS/MS)	RBC folate (nmol/L) intervention 682.7 (528.8–895.8), placebo 677.5 (548.2–807.6); serum folate (nmol/L) intervention 12.0 (9.9–14.0), placebo 10.8 (8.5–13.9)	Intervention 4.63% (4.60–4.66), placebo 4.60% (4.58–4.64)	No effect
**CANCER POPULATION**								
Cravo *et al.* 1994 [39]	22 patients with colorectal adenomas or carcinomas and 8 healthy controls	10 mg folic acid	6 months	Global DNA methylation in colorectal tissues	[^3^H] methyl incorporation	Serum folate (nmol/L) in intervention group 21.5 (3.8); placebo group 17.2 (3.6)	Normal-appearing rectal mucosa from controls 109000 dpm	Increased global DNA methylation
Cravo *et al.* 1998 [37]	20 colorectal adenoma patients	5 mg folic acid	6 months	Global methylation in colorectal tissues	[^3^H] methyl incorporation	Serum folate (nmol/L) 43.5 (8.3)	237039 dpm	No effect
Kim *et al.* 2001 [40]	20 colorectal adenoma patients	5 mg folic acid	1 year	Global DNA methylation in colorectal tissues	[^3^H] methyl incorporation	Serum folate (nmol/L) intervention 67.9, placebo 33.9; RBC folate (nmol/L) intervention 793.1, placebo 600.4	Intervention 220000 dpm;placebo 220000 dpm	Increased global DNA methylation
Pufulete *et al.* 2005 [41]	31 colorectal adenoma patients	400 µg folic acid	10 weeks	Global DNA methylation in leukocytes and colorectal tissues	[^3^H] methyl incorporation	Serum folate (nmol/L) intervention 16.7 (12.9–20.8), placebo 18.5 (14.9–22.2); RBC folate (nmol/L) intervention 639.0 (514.3–876.9), placebo 716.0 (591.4–840.6)	Leukocytes 748 (672–825) Bq/µg DNA; colon 602 (515–689) Bq/µg DNA	Increased global DNA methylation
Figueiredo *et al.* 2009 [38]	388 colorectal adenoma patients	1 mg folic acid	3 years	Global DNA methylation in colorectal tissues	Bisulfite pyrosequencing LINE-1 analysis	RBC folate (nmol/L) 898.2 (16.3); plasma folate (nmol/L) 20.9 (0.8)	None taken	No effect

1 conversion factor of 2.266 for folate from ng/mL to nmol/L.

On the other hand, our findings are unexpected, as dietary folate depletion/repletion studies in women have previously reported global DNA hypomethylation reversal following folate repletion [8], [9], [10]. In the United States, Shelnutt *et al.* conducted a separate folate feeding trial of young women aged 20 to 30, who were either MTHFR677CC or MTHFR677TT, participants were put on folate depletion diet of 115 µg dietary folate equivalents (DFE)/day for 7 weeks followed by a 7-week folate repletion diet of 400 µg DFE/day. Overall, global DNA methylation (measured using the liquid chromatography tandem mass spectrometry assay identical to the one we used in our study) decreased after folate depletion diet, but following folate repletion with folic acid supplementation, global DNA methylation significantly increased only in those with the TT genotype [42], [43]. In contrast with these findings, we did not find an effect of folic acid treatment according to genotype. As expected, baseline global DNA methylation in this American population was higher than baseline levels in our study, which was conducted in the Netherlands, where unlike the United States, folic acid fortification is not mandatory. Furthermore, baseline global DNA methylation in the Shelnutt *et al.* study was still higher than global DNA methylation in our population even after 3 years folic acid supplementation. This may be explained in part by the fact that our study population is substantially older than theirs and that their study only included women. Given that DNA hypomethylation seems to increase with age, reversion of DNA hypomethylation may be more difficult and may take longer in older individuals. Furthermore, there may be differences between the effects of folic acid supplementation (the synthetic form of folate) and natural dietary folate supplementation on DNA methylation, as folic acid and natural dietary folates enter the folate-mediated one-carbon metabolism cycle as different folate vitamers and may impact DNA methylation by uniquely altering metabolic flux of reactions in folate-mediated one-carbon metabolism.

In a study by Ingrosso *et al.* [43] that was partly designed to investigate the effects of folic acid administration on DNA methylation in men with hyperhomocysteinemia and uremia, DNA hypomethylation was higher in those with hyperhomocysteinemia compared to controls. Following supplementation with 15 mg of 5-methyltetrahydrofolate daily for 8 weeks, this DNA hypomethylation was reversed, which does not corroborate with our own findings. Although not a randomized controlled trial, the study conducted by Ingrosso *et al.* is insightful in presenting evidence of a lowering effect of the metabolically active form of folate on DNA methylation in men with hyperhomocysteinemia.

Our study is not without limitations. Firstly, we restricted the study population to those likely to benefit from folic acid supplementation; only those with elevated homocysteine were selected to participate in the FACIT trial since high homocysteine is a very sensitive indicator of low folate status. Intuitively, effects of folic acid supplementation are expected to be most pronounced in these individuals. We were not able to test whether effects are different in those with lower homocysteine concentrations. Genetic variability and other environmental factors and interactions between any of these also play a large role in folate-mediated one-carbon metabolism [44] and may subsequently influence DNA methylation [45]. Furthermore, while our results suggest that folic acid supplementation does not increase global DNA methylation, this does not preclude the possibility that folic acid supplementation has an effect on the methylation of specific individual genes, possibly in certain tissues. Folic acid supplementation may also have an effect on DNA methylation in subgroups of the population, such as those where polymorphisms from other folate-metabolizing genes have been observed to influence folate [46] and homocysteine [46], [47] concentrations.

Despite these limitations, our study has some noteworthy strengths. We were able to include two hundred sixteen participants in our analyses, making this the largest study to date investigating the effects of folic acid supplementation on global DNA methylation in healthy volunteers. Furthermore, we were able to carry out our study prospectively over three years, and this relatively long duration of folic acid supplementation can give us a better idea of the longer-term effects of folic acid.

In conclusion, our study shows that daily supplementation with 800 µg folic acid, while increasing folate status in blood, did not simultaneously alter long-term global DNA methylation in leukocytes of individuals with elevated homocysteine. The role of natural and synthetic folates in DNA methylation remains an area for further research. The relationship between leukocyte DNA methylation and DNA methylation in specific tissues should also be explored as well as genetic variability of folate-mediated one-carbon metabolism genes and other environmental factors that may have an impact on DNA methylation.
